# Supplementary material for: Genome Mining Discovery of a New Benzazepine Alkaloid Pseudofisnin A from the Marine Fungus Neosartorya pseudofischeri F27-1
Source: Antibiotics (Basel). 2022 Oct 20;11(10):1444. doi: 10.3390/antibiotics11101444 (PMC9598734; doi:10.3390/antibiotics11101444)

Supporting Information

Genome Mining Discovery of a New Benzazepine Alkaloid Pseudofisnin A from the Marine Fungus *Neosartorya pseudofischeri* F27-1

Xiao-Xin Xue[1], Lin Chen[1], and Man-Cheng Tang[1,2,*]

[1] State Key Laboratory of Microbial Metabolism, School of Life Sciences and Biotechnology, Shanghai Jiao Tong University, Shanghai 200240, China

[2] Zhangjiang Institute for Advanced Study, Shanghai Jiao Tong University, Shanghai 200240, China

*Correspondence: tangmc@sjtu.edu.cn

Table of contents

SUPPLEMENTART TABLES **S3**

Table S1. Bioinformatics analysis of the *pse* gene cluster. S3

Table S2. Primers used in this study. S3

Table S3. Plasmids used in this study. S4

Table S4. NMR data of nanangelenin B (**3**) in DMSO-d_6. .... S5

Table S5. NMR data of pseudofisnin B (**2**) in $CDCl_3$. S6

Table S6. NMR data of pseudofisnin A (**1**) in $CDCl_3$. S7

SUPPLEMENTART FIGURES **S8**

Figure S1. SDS-PAGE analysis of purified PseC. S8

Figure S2. Kinetic analysis of the methylation reaction catalyzed by PseC. S8

Figure S3. HR-ESIMS of (a) pseudofisnin B and (b) pseudofisnin A. S9

Figure S4. ^{1}H NMR spectrum of nanangelenin B (**3**) in DMSO-d_6. .... S100

Figure S5. ^{13}C NMR spectrum of nanangelenin B (**3**) in DMSO-d_6. .... S100

Figure S6. DEPT 135 spectrum of nanangelenin B (**3**) in DMSO-d_6. .... S111

Figure S7. ^{1}H NMR spectrum of pseudofisnin B (**2**) in $CDCl_3$. S111

Figure S8. ^{13}C NMR spectrum of pseudofisnin B (**2**) in $CDCl_3$. S122

Figure S9. DEPT 135 spectrum of pseudofisnin B (**2**) in $CDCl_3$. S122

Figure S10. COSY spectrum of pseudofisnin B (**2**) in $CDCl_3$. S133

Figure S11. HSQC spectrum of pseudofisnin B (**2**) in $CDCl_3$. S133

Figure S12. HMBC spectrum of pseudofisnin B (**2**) in $CDCl_3$. S134

Figure S13. ^{1}H NMR spectrum of pseudofisnin A (**1**) in $CDCl_3$. S134

Figure S14. ^{13}C NMR spectrum of pseudofisnin A (**1**) in $CDCl_3$. S135

Figure S15. DEPT 135 spectrum of pseudofisnin A (**1**) in $CDCl_3$. S135

Figure S16. COSY spectrum of pseudofisnin A (**1**) in $CDCl_3$. S136

Figure S17. HSQC spectrum of pseudofisnin A (**1**) in $CDCl_3$. S136

Figure S18. HMBC spectrum of pseudofisnin A (**1**) in $CDCl_3$. S13

SUPPLEMENTART TABLES

Table S1. Bioinformatics analysis of the *pse* gene cluster.

Genes	Proteins	Gene size (bp/aa)	Putative Function
*pse*A (XP_026618556)	PseA	7523/2381	NRPS
*pse*B (XP_026618558)	PseB	1315/415	Indoleamine 2,3-dioxygenase
*pse*C (XP_026618557)	PseC	1407/384	Methyltransferase
*pse*D (XP_026618560)	PseD	1236 /411	flavine-dependent monooxygenase
*pse*E (XP_026618559)	PseE	879/292	Hypothetical protein
*pse*F (XP_026618554)	PseF	387/128	Blasticidin-S deaminase
*pse*G (XP_026618553)	PseG	1185/394	UbiH type hydroxylase

Table S2. Primers used in this study.

Primer name	Primer sequence (5′→3′)
AmyB_5'-*pse*A-F	CCCTTCTCTGAACAATAAACCCCACAGAAGGCATTTATGGACAAC TGTAAGATGCCG
*pse*A-1-R	CAGACCACTCTGCACTGATAAG
*pse*A-2-F	CATCCAAGACGCATATCCTTG
*pse*A-2-R	AATATCGTCACACACCACGAC
*pse*A-3-F	CCATCATCGCATCTACAAGACG
glaA-*pse*A-R	CGACCAGTTCGGAAGATCAGGCTTGGTCCACTGAGGAAGATG
*pse*A-glaA-F	CATCTTCCTCAGTGGACCAAGCCTGATCTTCCGAACTGGTCG
*pse*F-glaA-R	CCTCGGGGGTGAGGGGCATTGCTGAGGTGTAATGATGCTG
glaA-*pse*F-F	CAGCATCATTACACCTCAGCAATGCCCCTCACCCCCGAGG
*pse*F-pYTP-R	GTGATGAGACCCAACAACCATGATACCAGGGGATTTGTTCTTCTTG AGCGTTGCACAG
glaA-*pse*D-F	CCTGAGCTTCATCCCCAGCATCATTACACCTCAGCAATGACGGTCA TCGACCAGGTC
AmyB-*pse*D_5'-R	GCTCGTTCGGCACCTTTAATCTCTCTGGAATGGACATACGCTC
*pse*D-AmyB_5'-F	GAGCGTATGTCCATTCCAGAGAGATTAAAGGTGCCGAACGAGC
*pse*B-AmyB_5'-R	GATCCCTCCGAGTCCTGGCATAAATGCCTTCTGTGGGGTTTATTG
AmyB_5'-*pse*B-F	CAATAAACCCCACAGAAGGCATTTATGCCAGGACTCGGAGGGATC
*pse*B-pYTU-R	ACAGTGGAGGACATACCCGTAATTTTCTGGGCATTTGGTCGACGAT GCCATTGATGTG
gpdA-*pse*E-F	ctaaccattaccccgccacatagacacatctaaacaATGTCGACAACTCCCACCACG
AmyB_5'-*pse*E-R	GCTCGTTCGGCACCTTTAATCAGCCAGCCTGCTTGAGTTGAAG
*pse*E-AmyB_5'-F	CTTCAACTCAAGCAGGCTGGCTGATTAAAGGTGCCGAACGAGC
*pse*C-AmyB_5'-R	CTGGCTAATTTCCCGTGTCATAAATGCCTTCTGTGGGGTTTATTG
AmyB_5'-*pse*C-F	CAATAAACCCCACAGAAGGCATTTATGACACGGGAAATTAGCCAG
glaA-*pse*C-F	CGACCAGTTCGGAAGATCAGGAGAAACATATGGTCATGGCTGC
*pse*C-glaA-F	GCAGCCATGACCATATGTTTCTCCTGATCTTCCGAACTGGTCG
*pse*G-glaA-R	CCTCGGGGGTGAGGGGCATTGCTGAGGTGTAATGATGCTG

glaA-*pse*G-F	CCAGCATCATTACACCTCAGCAATGCCCCAACCCCGAATCG
*pse*G-pYTR-R	CTAAAGGGTATCATCGAAAGGGAGTCATCCAATTTGTTGATCTGCG GTGAGAGATTG
*pse*A--pYTP-R	TGATGAGACCCAACAACCATGATACCAGGGGATTTCTTGGTCCAC TGAGGAAGATG
glaA-*pse*B-F	CTGAGCTTCATCCCCAGCATCATTACACCTCAGCAATGCCAGGACT CGGAGGGATC
*pse*B-pYTR-R	ACAGTGGAGGACATACCCGTAATTTTCTGGGCATTTGGTCGACGAT GCCATTGATGTG
gpdA-*pse*C-F	taaccattaccccgccacatagacacatctaaacaATGACACGGGAAATTAGCCAG
*pse*C-pYTU-R	CTAAAGGGTATCATCGAAAGGGAGTCATCCAATTTAGAAACATATG GTCATGGCTGC
pET28a-*pse*C-F	GTGCCGCGCGGCAGCCATATGATGACACGGGAAATTAGCCAG
*pse*C-1-R	CCTCCCGCAACGCACATCTGAGTAGGGACCTTGAAGCC
*pse*C-2-F	GGCTTCAAGGTCCCTACTCAGATGTGCGTTGCGGGAGG
*pse*C-2-R	GTCATGAGACCAACATCGGACATATGATGGACCCACGCGCCTC
*pse*C-3-F	GAGGCGCGTGGGTCCATCATATGTCCGATGTTGGTCTCATGAC
*pse*C-3-R	GAAAGCGTAGAAAGATTGGTCCGTCCGGTACGCCATCTGAAACG
*pse*C-4-F	CGTTTCAGATGGCGTACCGGACGGACCAATCTTTCTACGCTTTC
*pse*C-4-R	CCTAGGATGGTGCAACAGTCCTCTGAAGTATGGTCCATCAGGATG
*pse*C-5-F	CATCCTGATGGACCATACTTCAGAGGACTGTTGCACCATCCTAGG
pET28a-*pse*C-R	ACGGAGCTCGAATTCGGATCCCTAGCCCACTCTCTTCAGAGC

Table S3. Plasmids used in this study.

**Plasmids**	**Vector**	**Genes**
pXXX1-1	pYTP	AmyB_5'-*pse*A; glaA-*pse*F
pXXX1-2	pYTU	AmyB_5'-*pse*B; glaA-*pse*D
pXXX1-3	pYTR	GpdA-*pse*E; AmyB_5-*pse*C; GlaA-*pse*G
pXXX1-4	pYTP	AmyB_5'-*pse*A
pXXX1-5	pYTU	glaA-*pse*B
pXXX1-6	pYTR	GpdA-*pse*C
pXXX1-7	pET28a	T7p-N-his-*pse*C-T7t

Table S4. NMR data of nanangelenin B (**3**) in DMSO-d_6.

^{1}H NMR spectrum (600 MHz), ^{13}C NMR spectrum (150 MHz)

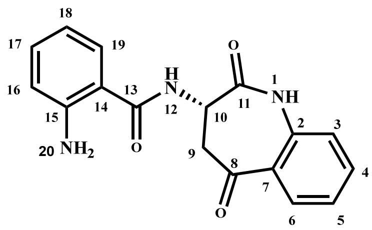

nanangelenin B (3)

No.	δ_H (J in Hz)	δ_C, type
1	10.36, s	
2		137.7, C
3	7.26, t (7.8)	124.3, CH
4	7.61, m	134.2, CH
5	7.19, d (8.1)	122.2, CH
6	7.75, dd (7.9, 1.7)	130.0, CH
7		128.4, C
8		197.8, C
9	3.02, dd (18.7, 2.7)<br>3.26, dd (18.7, 13.3)	45.8, CH_2
10	5.00, ddd (13.3, 7.5, 2.7)	46.1, CH
11		171.2, C
12	8.42, d (7.4)	
13		168.6, C
14		113.9, C
15		149.7, C
16	6.70, d (8.2)	116.4, CH
17	7.16, m	132.1, CH
18	6.54, t (7.5)	114.6, CH
19	7.58, dd (8.0, 1.6)	128.4, CH
20	6.36, brs	

“m” means overlapped or multipet with other signals.

The NMR data of 1 is identical to the data reported in *J. Am. Chem. Soc.* 2020, 142(15), 7145-7152.

Table S5. NMR data of pseudofisnin B (**2**) in $CDCl_3$.
^{1}H NMR spectrum (600 MHz), ^{13}C NMR spectrum (150 MHz)

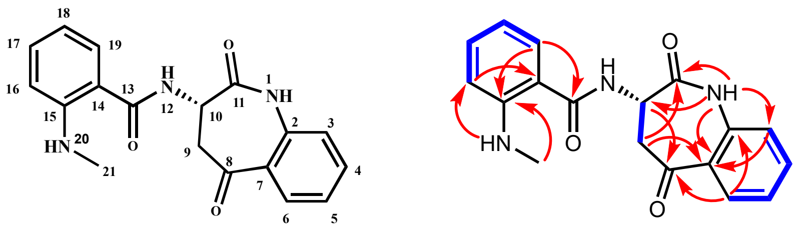

No.	δ_H (J in Hz)	δ_C, type
1	7.70, s	
2		135.4, C
3	7.00, d (8.0)	122.1, CH
4	7.56, t (7.7)	134.4, CH
5	7.32, t (7.7)	126.2, CH
6	7.93, d (8.0)	131.2, CH
7		129.8, C
8		196.6, C
9	3.04, dd (18.9, 12.9)<br>3.44, m	47.3, CH_2
10	5.20, m	46.5, CH
11		171.7, C
12		
13		169.0, C
14		113.5, C
15		150.9, C
16	6.67, d (8.47)	111.3, CH
17	7.35, t (7.70)	133.5, CH
18	6.63, t (7.70)	114.6, CH
19	7.49, d (8.07)	127.7, CH
20	6.80, s	
21	2.85, d (4.30)	29.6, CH_3

"m" means overlapped or multipet with other signals.

Table S6. NMR data of pseudofisnin A (**1**) in $CDCl_3$.
^{1}H NMR spectrum (600 MHz), ^{13}C NMR spectrum (150 MHz)

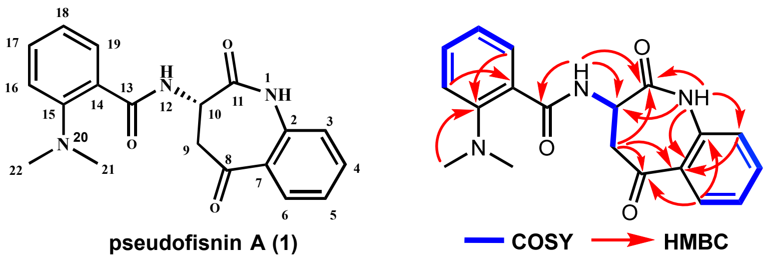

No.	δ_H (J in Hz)	δ_C, type
1	7.69, s	
2		135.7, C
3	6.99, d (8.0)	122.0, CH
4	7.54, t (7.7)	134.3, CH
5	7.31, t (7.7)	126.0, CH
6	7.93, d (8.0)	131.1, CH
7		130.1, C
8		197.5, C
9	3.07, dd (19.0, 12.9)<br>3.47, dd (19.0, 3.2)	47.4, CH_2
10	5.39, m	47.0, CH
11		171.9, C
12	11.06, d (6.30)	
13		165.8, C
14		126.7, C
15		153.0, C
16	7.27, d (8.0)	120.3, CH
17	7.45, t (7.7)	132.4, CH
18	7.19, t (7.7)	124.3, CH
19	8.11, d (7.9)	131.4, CH
20		
21	2.79, s	45.4, CH_3
22	2.79, s	45.4, CH_3

"m" means overlapped or multipet with other signals.

SUPPLEMENTART FIGURES

Figure S1. SDS-PAGE analysis of purified PseC.

Left lane: protein marker; Right lane: the purified PseC (46.8 kDa).

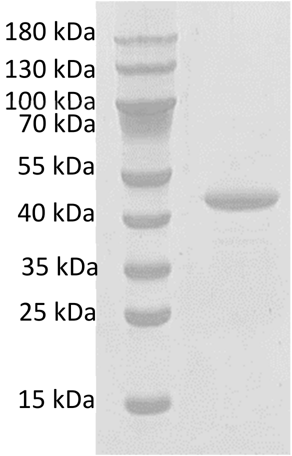

Figure S2. Kinetic analysis of the methylation reaction catalyzed by PseC.

(a) nanangelenin B (**3**) as the substrate; (b) pseudofisnin B (**2**) as the subsrate.

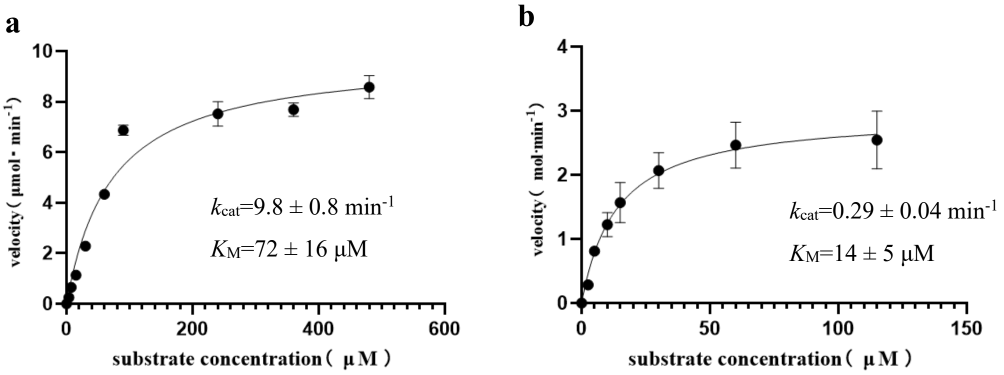

Figure S3. HR-ESIMS data of (a) pseudofisnin B and (b) pseudofisnin A.

a

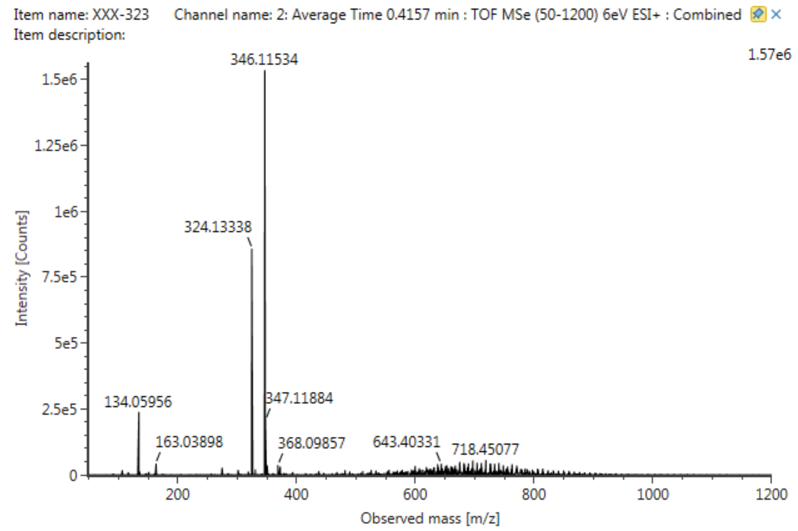

b

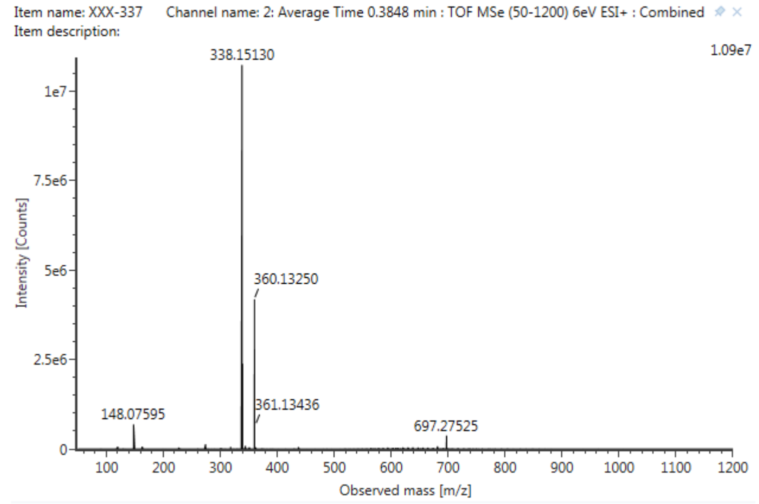

Figure S4. ^{1}H NMR spectrum of nanangelenin B (**3**) in DMSO-d_6.

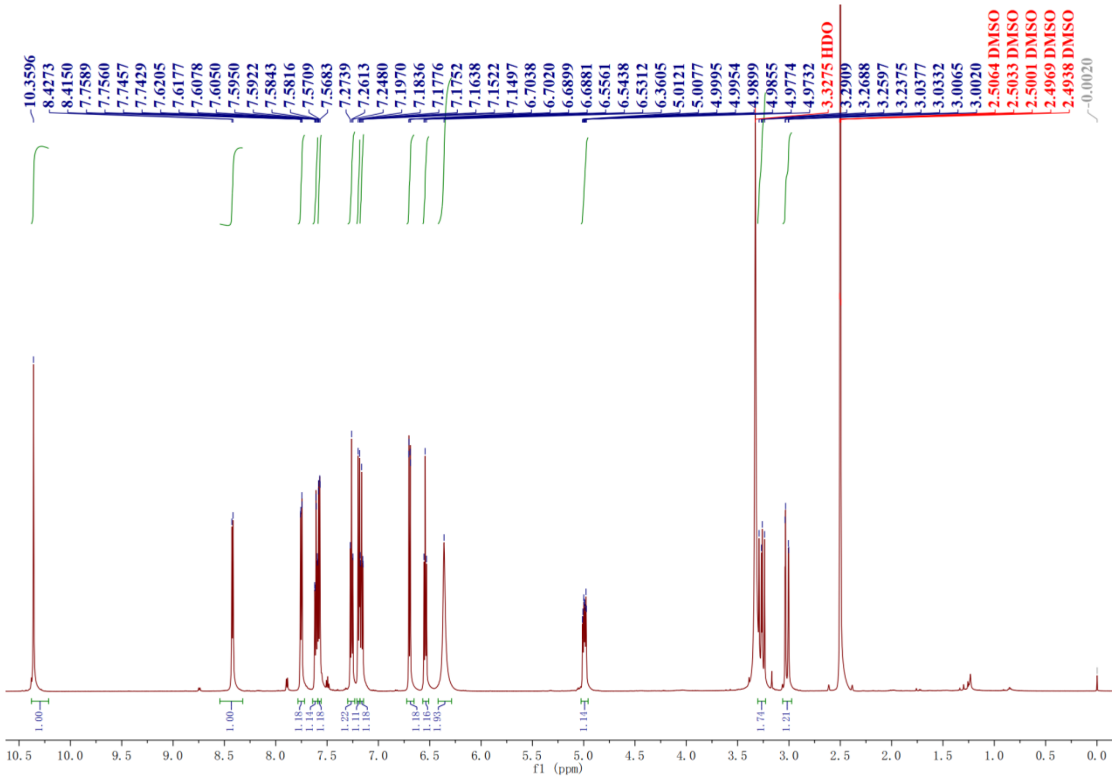

Figure S5. ^{13}C NMR spectrum of nanangelenin B (**3**) in DMSO-d_6.

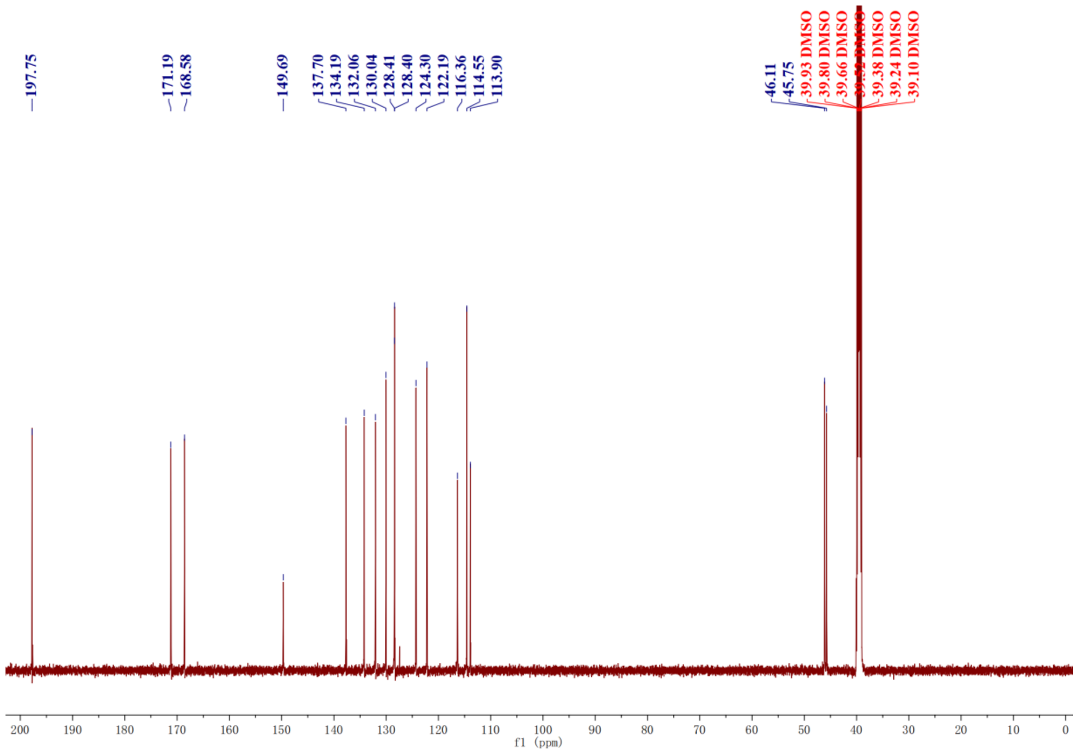

Figure S6. DEPT 135 spectrum of nanangelenin B (**3**) in DMSO-d_6.

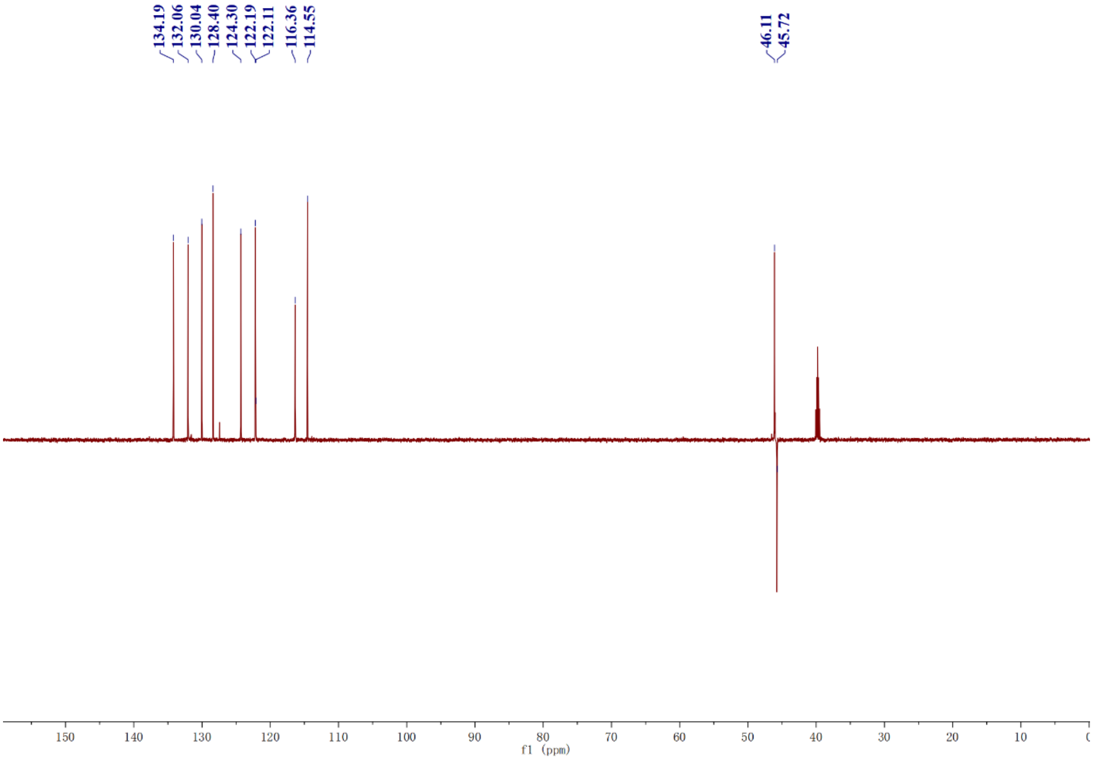

Figure S7. ^{1}H NMR spectrum of pseudofisnin B (**2**) in $CDCl_3$.

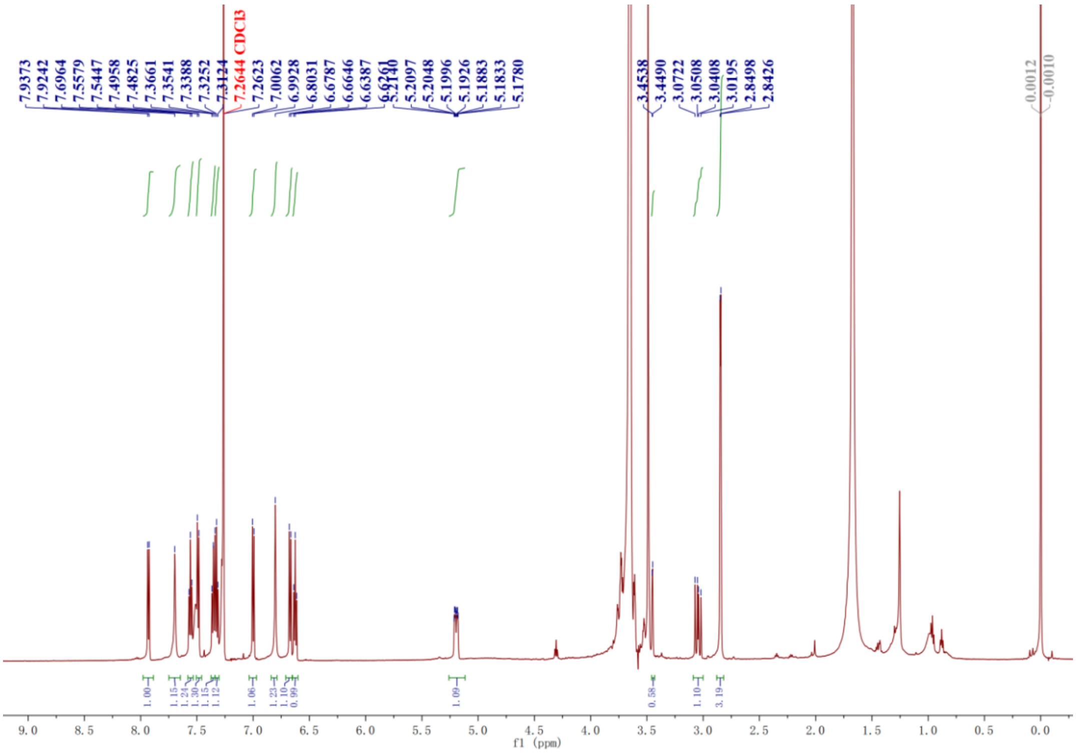

Figure S8. ^{13}C NMR spectrum of pseudofisnin B (**2**) in $CDCl_3$.

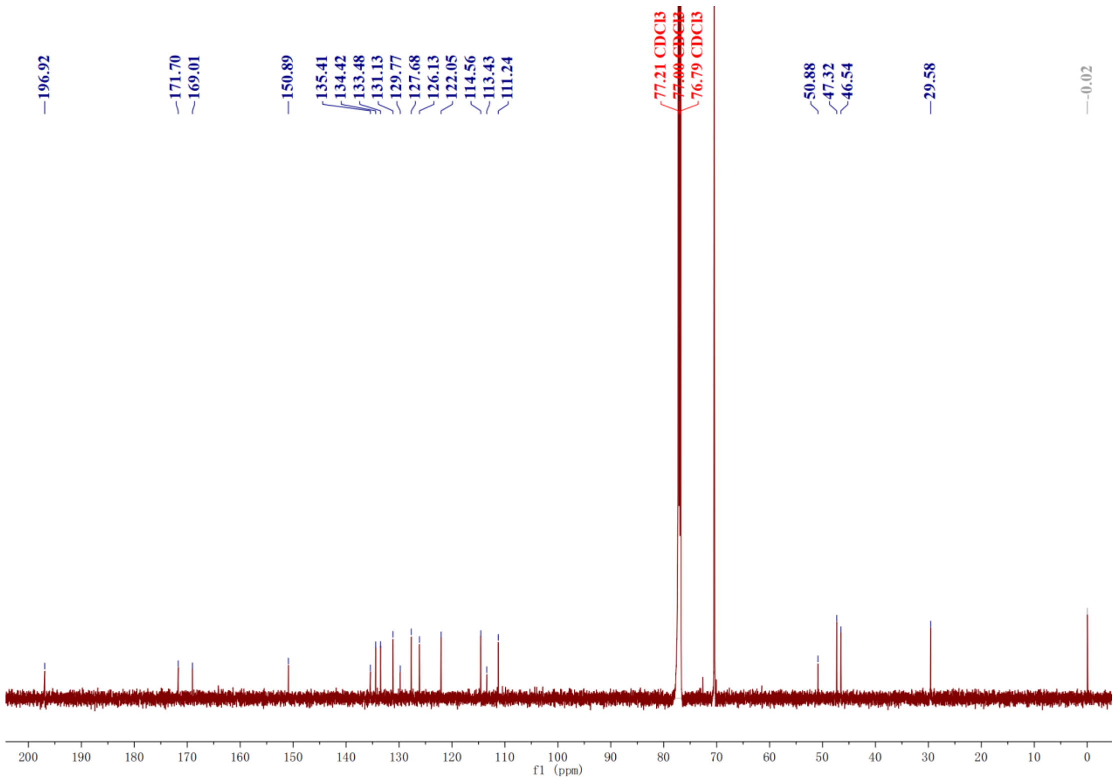

Figure S9. DEPT 135 spectrum of pseudofisnin B (**2**) in $CDCl_3$.

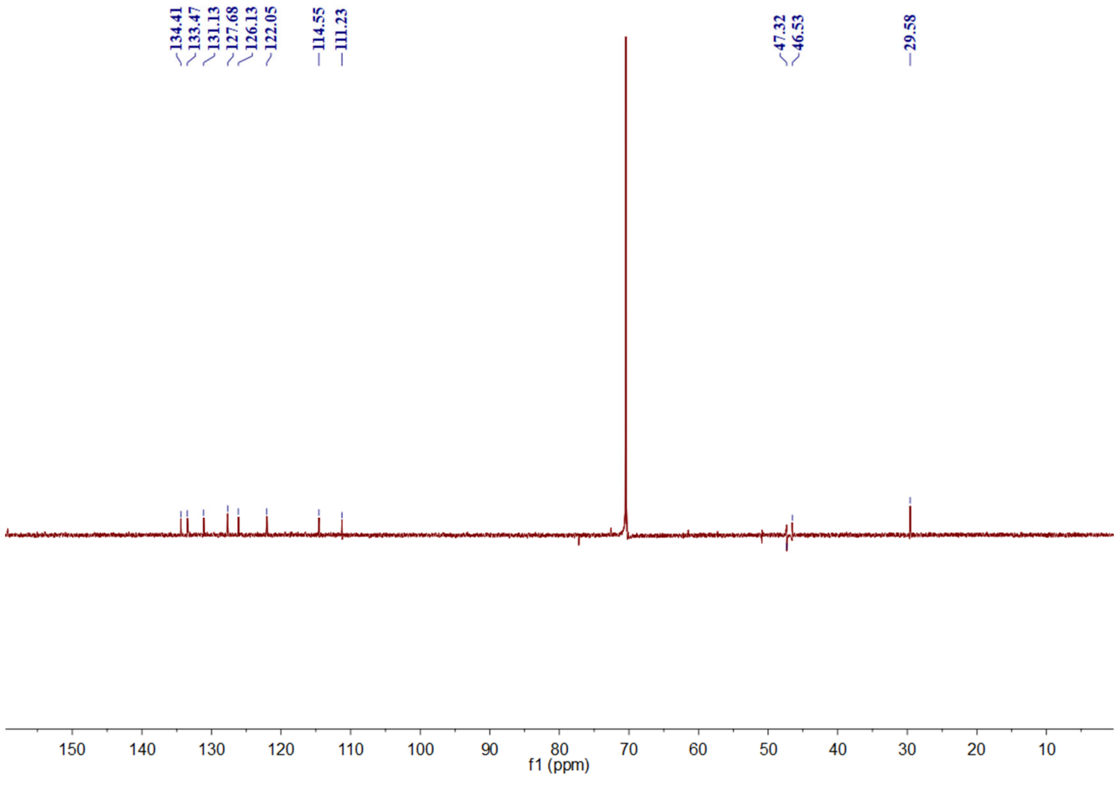

Figure S10. COSY spectrum of pseudofisnin B (**2**) in $CDCl_3$.

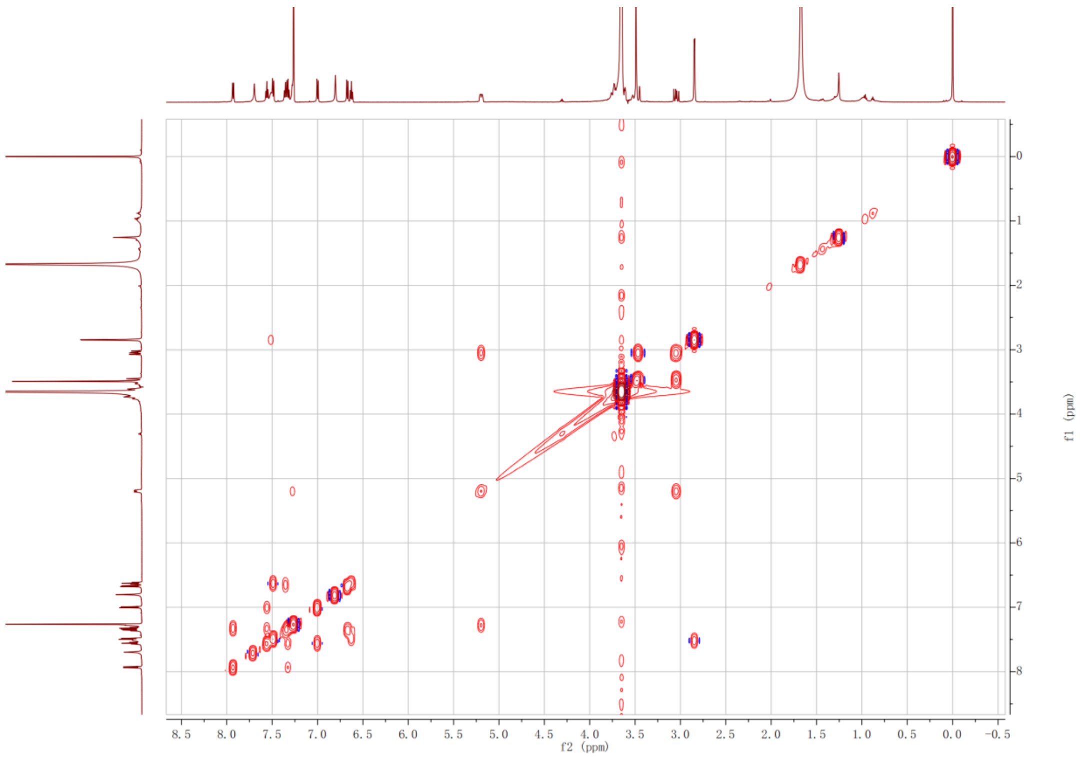

Figure S11. HSQC spectrum of pseudofisnin B (**2**) in $CDCl_3$.

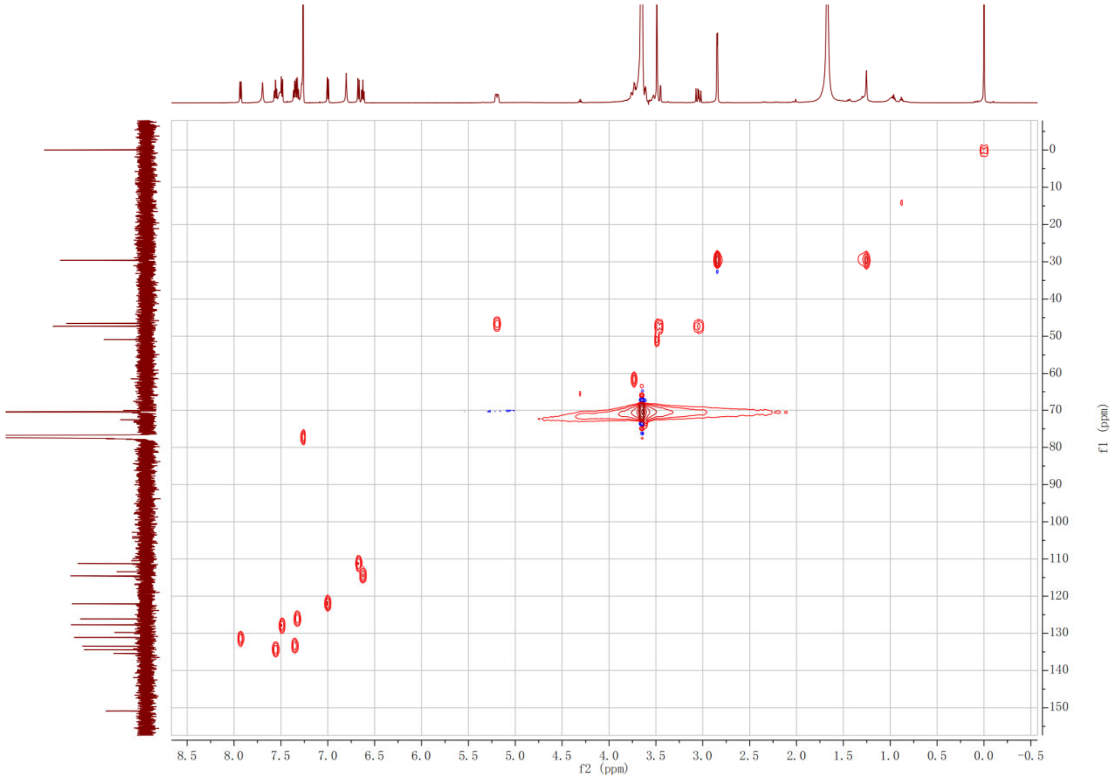

Figure S12. HMBC spectrum of pseudofisnin B (**2**) in $CDCl_3$.

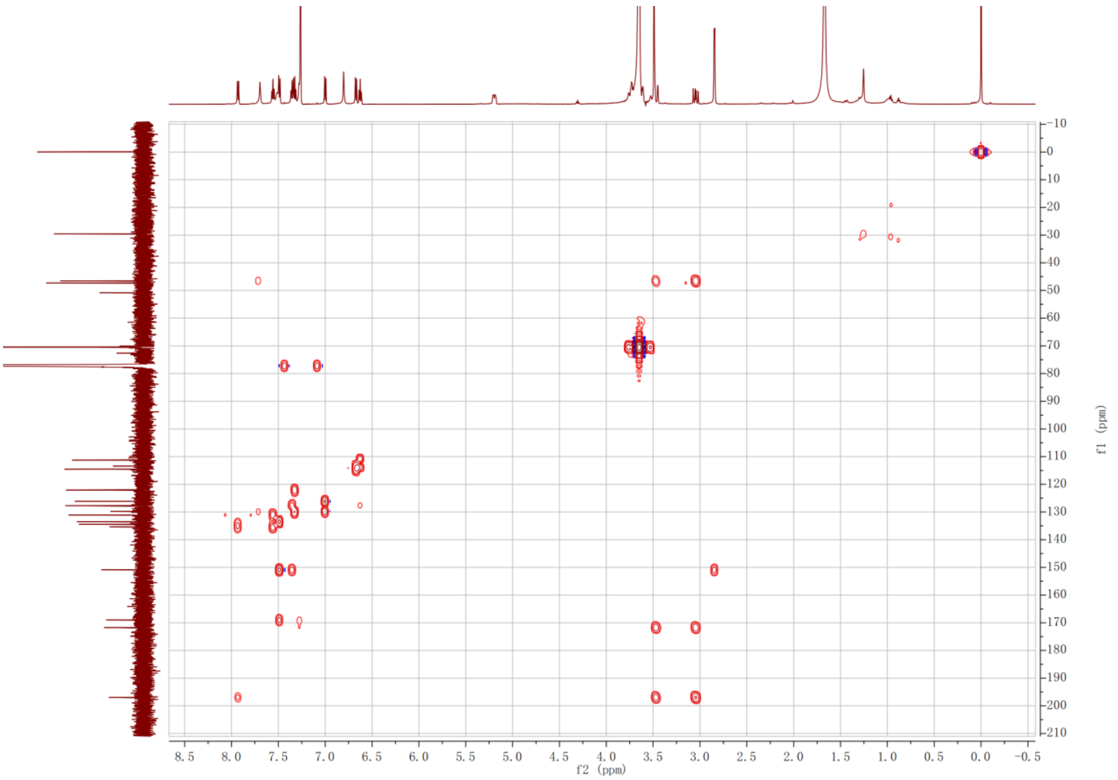

Figure S13. ^{1}H NMR spectrum of pseudofisnin A (**1**) in $CDCl_3$.

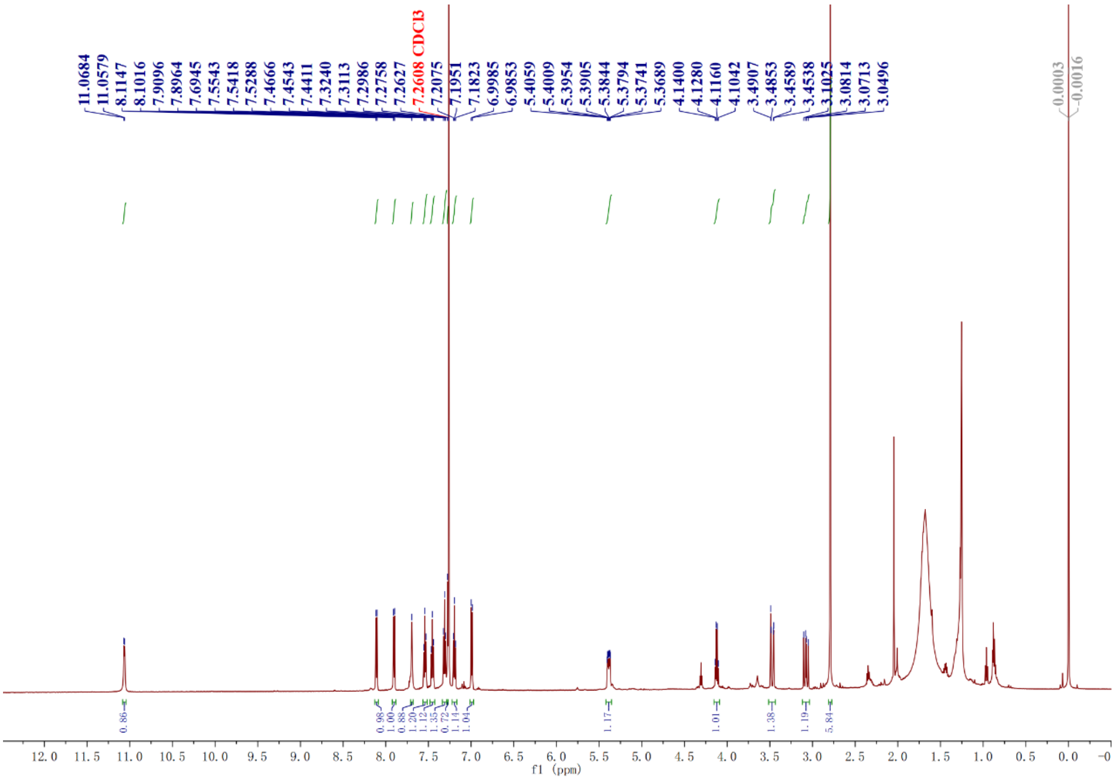

Figure S14. ^{13}C NMR spectrum of pseudofisnin A (**1**) in $CDCl_3$.

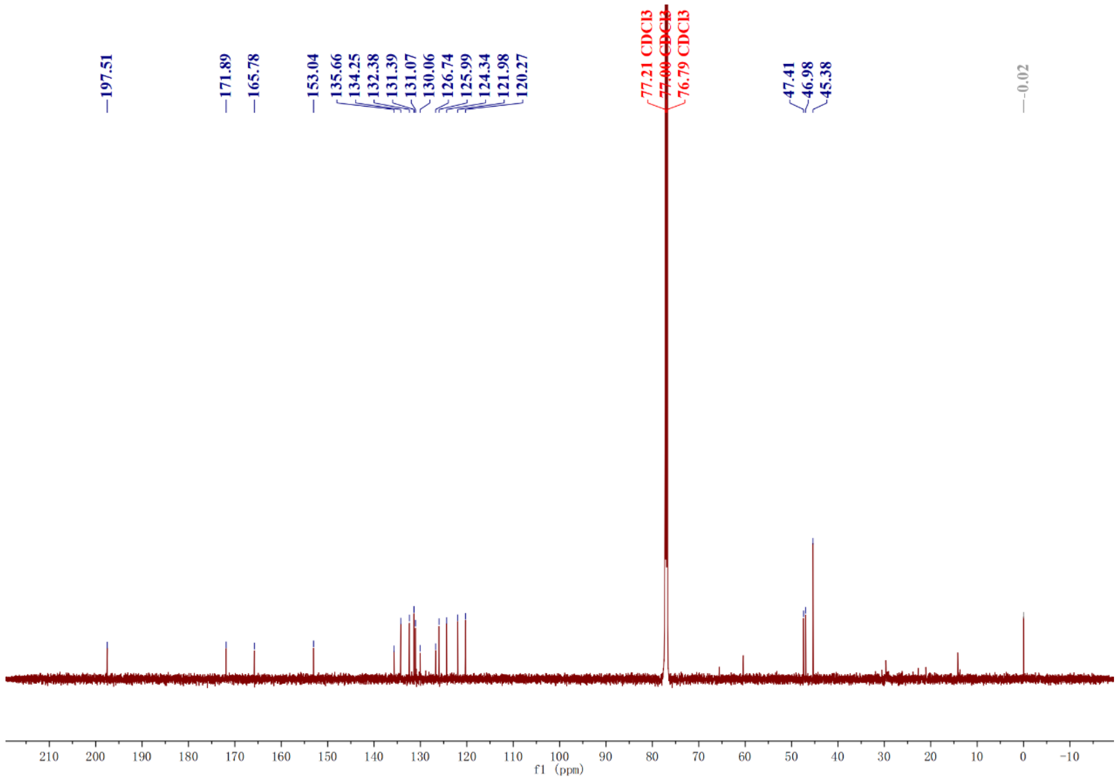

Figure S15. DEPT 135 spectrum of pseudofisnin A (**1**) in $CDCl_3$.

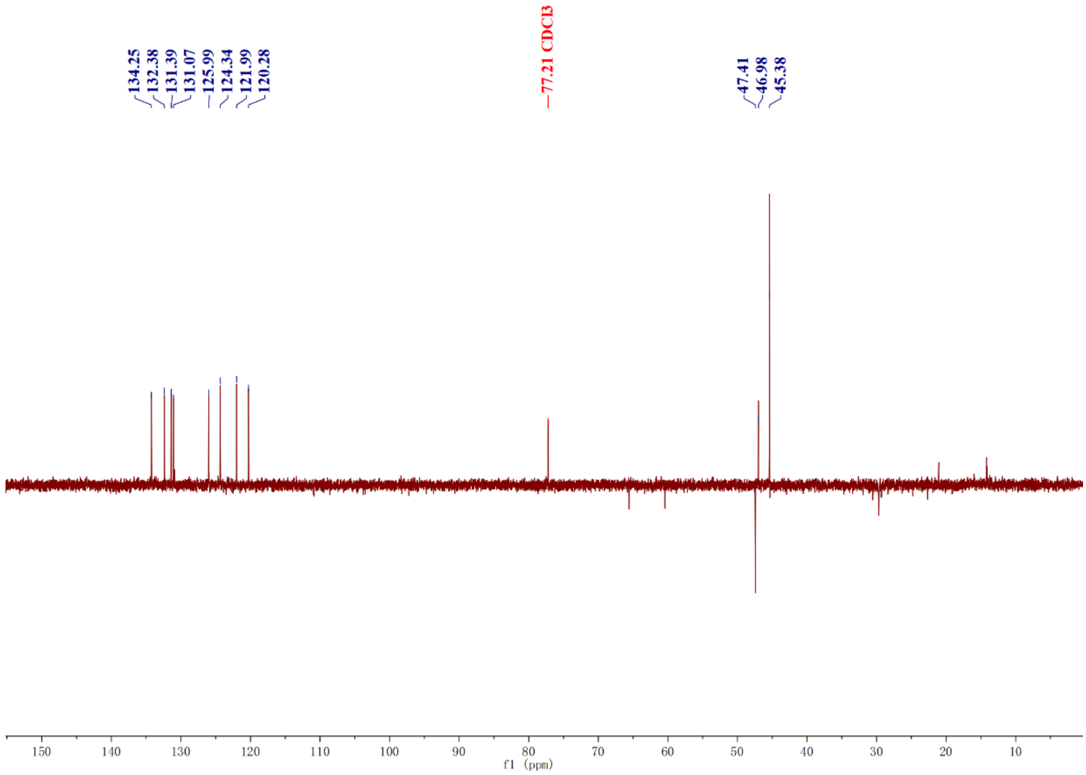

Figure S16. COSY spectrum of pseudofisnin A (**1**) in $CDCl_3$.

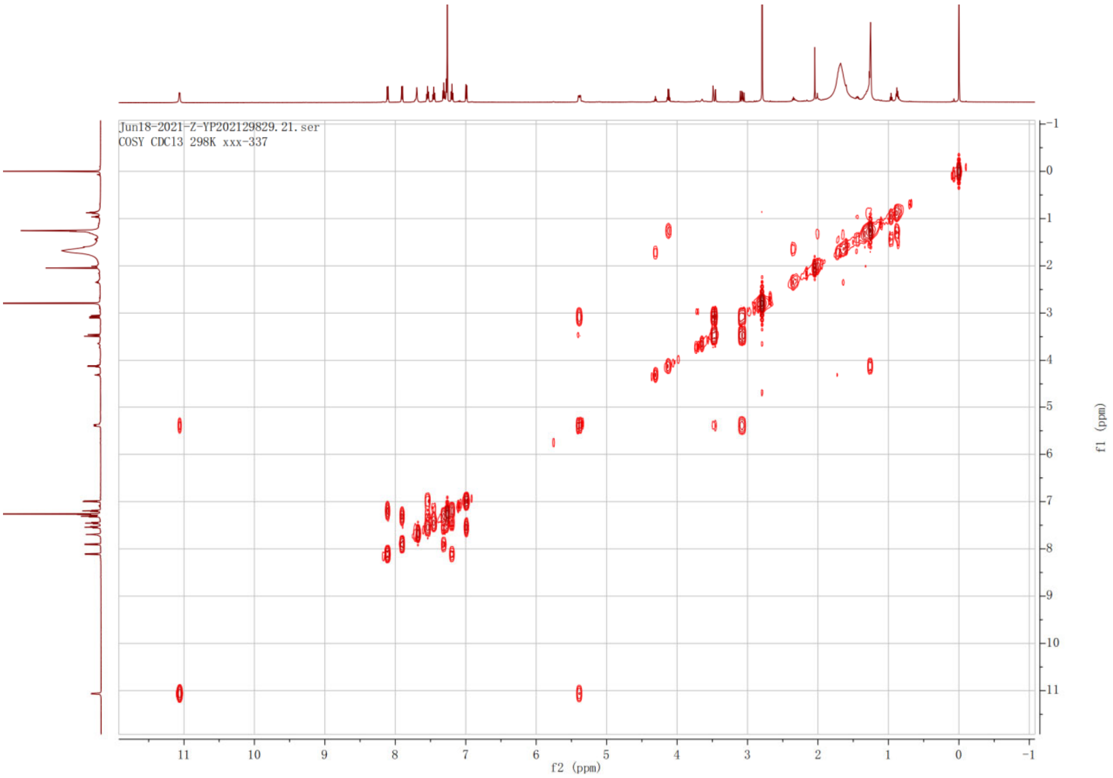

Figure S17. HSQC spectrum of pseudofisnin A (**1**) in $CDCl_3$.

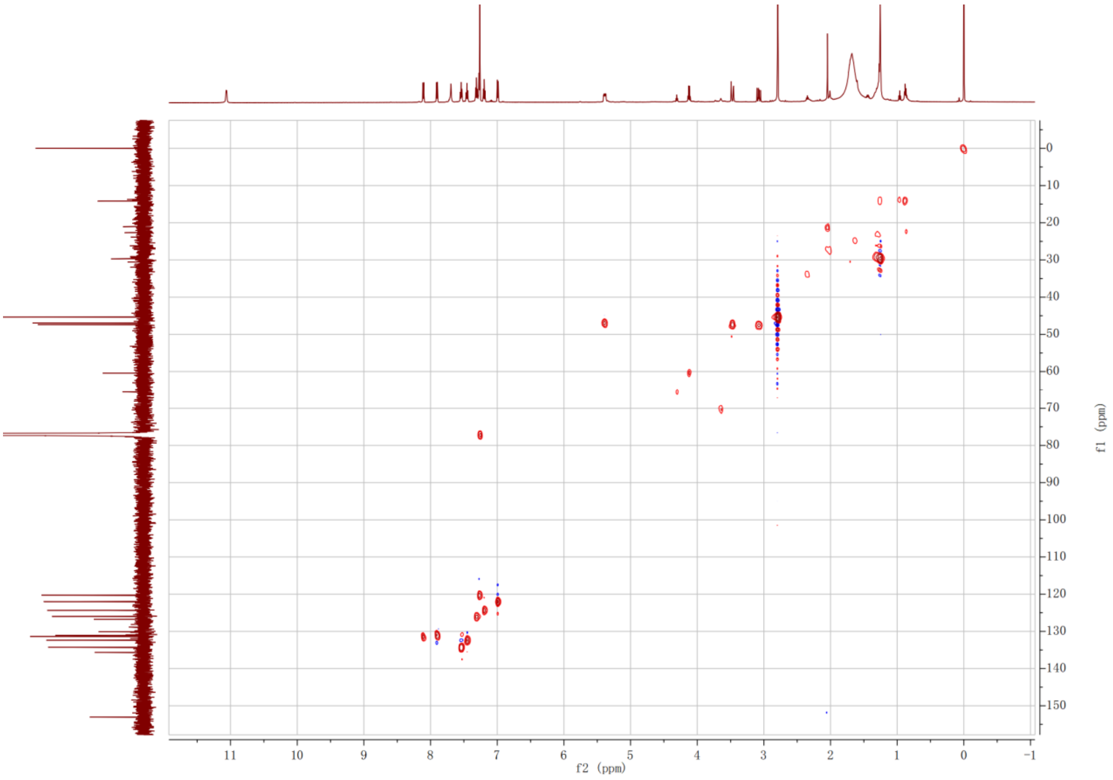

Figure S18. HMBC spectrum of pseudofisnin A (**1**) in $CDCl_3$.

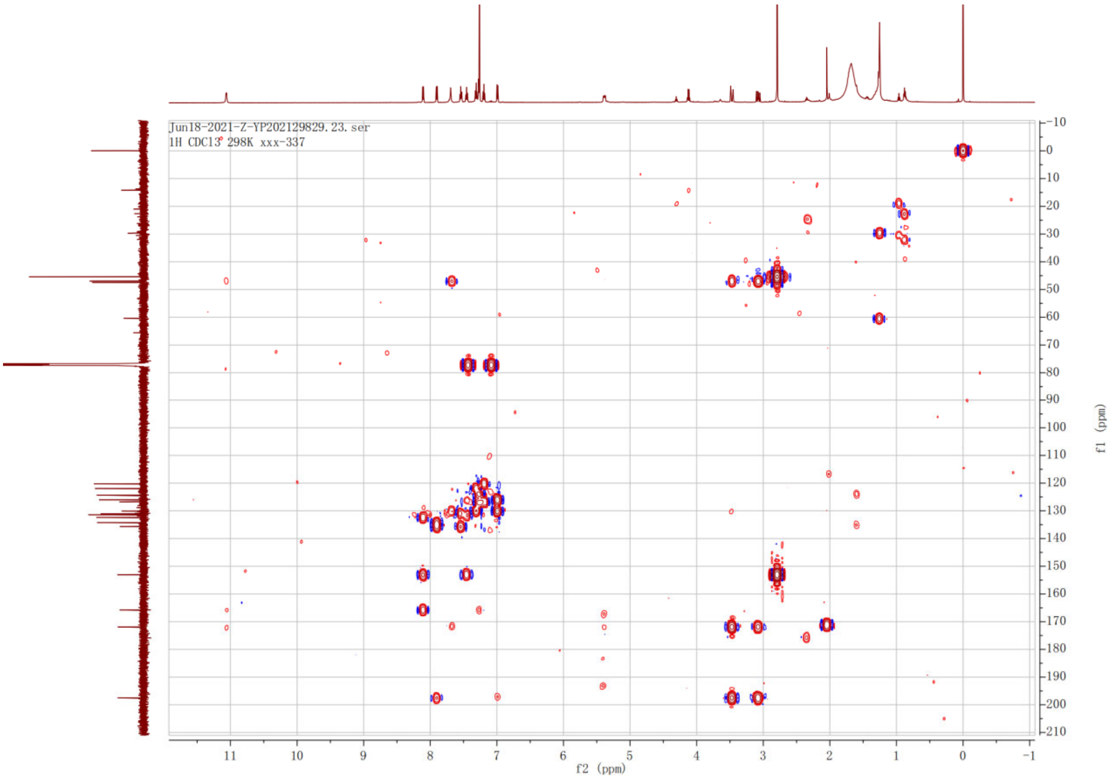

Supplement: Supplementary file 1 [file antibiotics-11-01444-s001.zip › antibiotics-1975547-supplementary.pdf]
